# Factors Associated with Complications of Snakebite Envenomation in Health Facilities in the Cascades Region of Burkina Faso from 2016 to 2021

**DOI:** 10.3390/tropicalmed9110268

**Published:** 2024-11-06

**Authors:** Rene Kinda, Sidikiba Sidibe, Dramane Zongo, Tieba Millogo, Alexandre Delamou, Seni Kouanda

**Affiliations:** 1 National Malaria Research and Training Centre (CNRFP), Ouagadougou 01 BP 2208, Burkina Faso; 2 African Centre of Excellence in the Prevention and Control of Communicable Diseases (CEA-PCMT), Faculty of Sciences and Health Techniques, Gamal Abdel Nasser University of Conakry, Conakry G8WF, Guinea; ssidibe@maferinyah.org (S.S.); adelamou@gmail.com (A.D.); 3 The African Institute of Public Health (AIPH), Ouagadougou 12 BP 50, Burkina Faso; zdramane@gmail.com (D.Z.); millogorod@gmail.com (T.M.); senikouanda@gmail.com (S.K.); 4 The Institut de Recherche en Sciences de la Santé, Ouagadougou 03 BP 7192, Burkina Faso

**Keywords:** snakebite, envenomation, complications

## Abstract

Snakebite envenomation remains a major cause of morbidity and mortality in rural populations. This study identified factors associated with the complications of snakebite envenomation reported in the Cascades region of Burkina Faso. This cross-sectional study used the routine data of patients admitted for snakebite envenomation at five health facilities between 1 January 2016 and 31 December 2021. Data were collected on sociodemographic, clinical, and therapeutic characteristics of patients with signs of envenomation. Bivariate and multivariate analyses were conducted to identify factors associated with complications. Among the 846 patients with envenomation, 355 (42%) experienced complications. Local complications (23.2%, 196/846) included wounds and skin necrosis, whereas systemic complications (34.3%, 290/846) included hemorrhage, shock, and coma. Of all complicated cases, 7.6% (27/355) died. Factors associated with complications were rural residence (AOR: 4.80; 95% CI: 2.21–11.4), incision at the bite site (AOR: 4.31; 95% CI: 2.51–7.52), tourniquet application (AOR: 5.52; 95% CI: 1.42–30.8), bleeding (AOR: 14.2; 95% CI: 8.80–23.4), abnormal vital signs (AOR: 14.3; 95% CI: 9.22–22.7), and lack of antivenom administration (AOR: 2.92; 95% CI: 1.8–4.8). These findings highlight the importance of antivenom availability and public awareness for reducing the incidence of complications of snakebite envenomation.

## 1. Introduction

The complications of snakebite envenomation constitute a serious public health problem, causing chronic disabilities, irreversible kidney failure, and death, particularly in sub-Saharan Africa [1,2]. Globally, snakebites result in an estimated 2.7 million envenomation and 81,000 to 138,000 deaths annually, with sub-Saharan Africa bearing a significant portion of this burden [3]. People who have experienced snakebites typically present to health facilities with signs of local and/or systemic complications [4]. The people most at risk are those who are poor, live in remote areas, and have limited access to health care.

Assessing the true extent of complications of snakebite envenomation is challenging because of a lack of reliable statistics. However, various studies have documented the frequency of complications and their lethal consequences. Hemorrhagic complications are the most frequently reported (78.9%), and fatal outcomes occur in 7.8% of bites [5,6]. In Burkina Faso, between 2010 and 2014, the annual incidence and mortality rates were 130 snakebites and 1.75 deaths per 100,000 people, respectively [7]. Despite these statistics, few studies have investigated the factors associated with complications of snakebite envenomation in Burkina Faso.

In the Cascades region, the most commonly encountered snake species are vipers (*Echis ocellatus*) and cobras (*Naja nigricollis*). These species are known for their potent venom, which can cause severe local and systemic complications [8].Complications and deaths often result from delayed access to care given that victims initially seek traditional treatments and only later present at health facilities with advanced complications [4]. The use of tourniquets accelerates the necrosis of affected limbs. In addition, access to quality serum antivenom is frequently limited [5,9]. Most studies conducted in Burkina Faso have focused on the prevalence or incidence of snakebites. Recent research on complications has been conducted using case reports from referral hospitals [7,10,11,12,13,14]. Few studies have explored both the complications of snakebite envenomation and associated factors.

This study aims to analyze the factors associated with complications of snakebite envenomation using data recorded at five health facilities in the Cascades region, one of the most affected areas in Burkina Faso [7]. The results could help the National Program for the Control of Neglected Tropical Diseases strengthen snakebite prevention measures and improve the management of envenomation.

## 2. Materials and Methods

### 2.1. Study Design

This cross-sectional study used secondary data obtained from a review of consultation registers and patient medical records.

### 2.2. Study Setting

Burkina Faso comprises 13 health regions, including the Cascades region, located in the southwest of the country. The vegetation of the region is Guinean savanna, which is home to various types of snakes, such as vipers and cobras [10,15,16]. According to the latest general population and housing census, the population of the Cascades region was estimated at 812,466 inhabitants in 2019 [17]. The Cascades region is subdivided into three health districts (Banfora, Mangodara, and Sindou) with a regional referral hospital. The study was conducted in the Banfora and Sindou health districts, involving four health facilities (Sindou Medical Center with a surgical unit, Niangoloko Medical Center, Niankorodougou Medical Center, and Yendere Health and Social Promotion Center) and the Banfora Regional Hospital Center. These facilities were selected for their relevance to the study, some due to their pivotal position in the region’s health care network, and others for regularly reporting cases of snakebite envenomation.

### 2.3. Study Population

**Figure 1:**
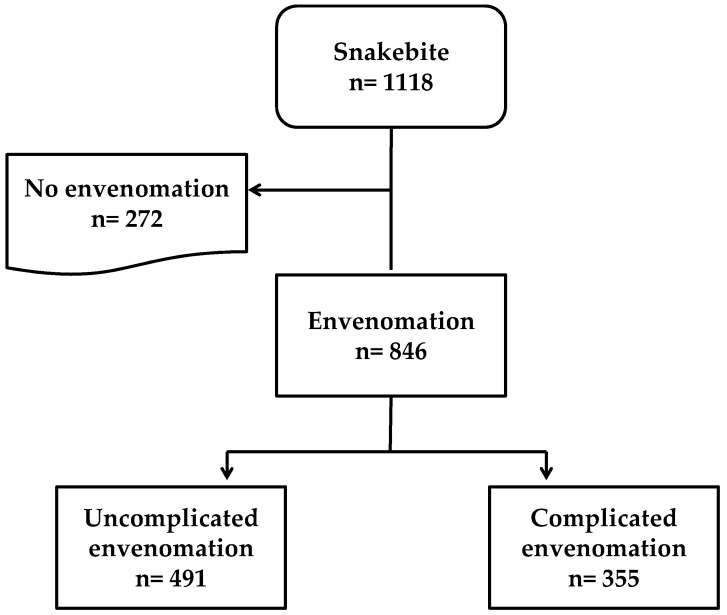
Study flow chart. Snakebite cases admitted to five health facilities in the Cascades region of Burkina Faso, 2016–2021.

We included all individuals who consulted the selected health facilities for snakebites between 1 January 2016 and 31 December 2021. The analysis focused on individuals with snakebite and envenomation admitted to these facilities during the study period (*n* = 846, Figure 1).

### 2.4. Data Collection

Variables related to the study objectives were extracted from consultation registers and patient medical records using a questionnaire developed using KoBoCollect 1.4.8 (1057) software. Data were collected from 29 September to 15 November 2022 in health facilities.

### 2.5. Measurement of Variables

Envenomation by snakes involves venom injection, and it causes various harmful effects, such as tissue damage, systemic toxicity, and symptoms, depending on the specific venom of the snake species. Envenomation can lead to serious medical emergencies requiring prompt intervention like antivenom treatment. Hemotoxicity refers to the toxic effects of snake venom on blood, causing disrupted circulation, blood loss, vessel damage, and cell destruction. The symptoms include uncontrolled bleeding and organ damage. Neurotoxicity refers to the toxic effects of snake venom on the nervous system, leading to symptoms such as muscle weakness, paralysis, difficulty breathing, and palpebral ptosis. Cytotoxicity was defined as pain, swelling, blistering, and tissue necrosis around the bite area. Venom substances directly damage and destroy cells, leading to local tissue damage at the bite site.

The dependent variable was the occurrence of snakebite envenomation complications, defined by the presence of hemotoxic, neurotoxic, or cytotoxic symptoms and signs. These corresponded to either local complications (presence of cytotoxic signs) or systemic complications (presence of hemotoxic or neurotoxic signs) requiring immediate management [18]. This binary variable is 1 and 0 and indicates complicated and uncomplicated envenomation, respectively.

The independent variables included sociodemographic (age, sex, occupation, residence), clinical (presence of comorbidity, initial treatment, and course of the disease), and therapeutic (administration of serum antivenom) characteristics, which were collected from consultation registers and medical records.

### 2.6. Statistical Analysis

Data collected from the consultation registers and patient medical records were analyzed using R 4.2.1 software. The characteristics of people who experienced snakebite envenomation are presented as frequencies, percentages, or medians with interquartile ranges. Bivariate analysis was performed using the chi-square test to explore factors associated with snakebite envenomation-related complications. The results are presented as odds ratios (ORs) and 95% confidence intervals (CIs). Variables with a *p*-value of ≤0.20 in the bivariate analysis were included in the multivariate logistic regression model to calculate adjusted odds ratios (AOR) with 95% CI. The variables included in this model were derived from the study database and literature review. Differences were considered statistically significant with *p* ≤ 0.05.

## 3. Results

Figure 1 shows the flow chart of snakebite cases to envenomation and complicated envenomation at the five health facilities in the Cascades region of Burkina Faso from 2016 to 2021. Overall, 846 patients presented with envenomation.

### 3.1. Sociodemographic and Clinical Characteristics

#### 3.1.1. Sociodemographic Characteristics

**Figure 2:**
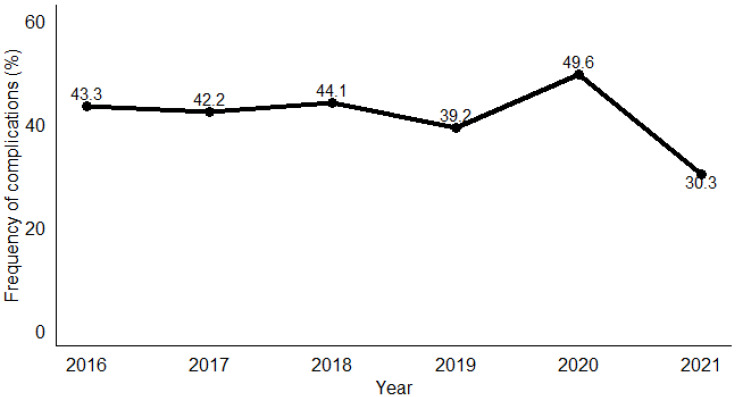
Annual frequency of complications related to snakebite envenomation in the Cascades region of Burkina Faso, 2016–2021.

The sociodemographic characteristics of the 846 individuals with envenomation are presented in Table 1. The median age of envenomation victims was 17 years (IQR = 10–32). Children under 15 years of age (369/846, 43.6%) were the most affected by snakebite envenomation. Most victims were male (54.6%) and lived in rural areas (88.2%). Envenomation was recorded throughout the year, with 49.2% reported in the first half (January–June) and 50.8% in the second half (July–December) (Figure 2).

#### 3.1.2. Clinical Characteristics

Of the 846 patients, 102 had information on snake species. Among them, 88 (86.3%) had viper bites and 14 (13.7%) had cobra bites. Patients primarily identified the species based on their descriptions of the snakes’ appearance and behavior. Table 2 shows the clinical characteristics of cases of snakebite envenomation. Regarding the first procedures performed at home before being taken to the hospital, the bite site was incised (201/846, 23.8%), a black stone was used (118/846, 13.9%), and a tourniquet was applied (67/846, 7.9%) in patients with snakebite envenomation. Local signs such as edema and pain at the bite site were present in the vast majority (811/846, 95.9%) of snakebite envenomation victims. The most common signs were hemotoxicity (275/846, 32.5%), with bleeding often accompanied by dyspnea (95/846, 11.2%). Comorbidities (50/846, 5.9%), such as severe malaria and hypertension, were also present. Regarding treatment, 69.7% (590/846) of envenomation victims received serum antivenom from health facilities.

### 3.2. Frequency of Complications of Snakebite Envenomation

**Figure 3:**
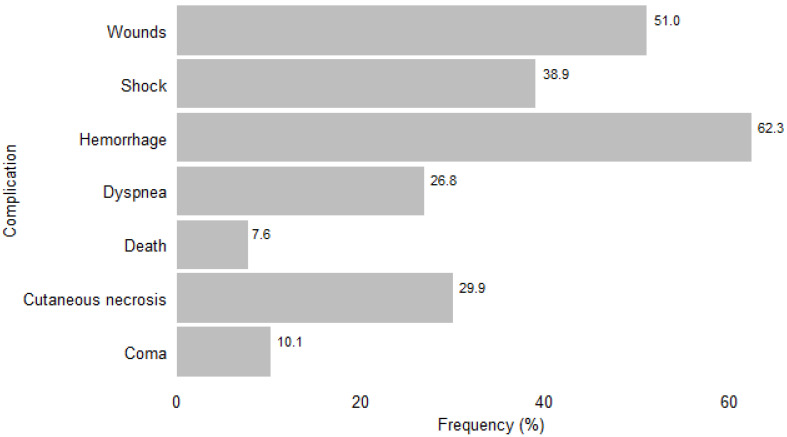
Types of complications of snakebite envenomation (%) in the Cascades region of Burkina Faso, 2016–2021 (*n* = 355).

Overall, complications were observed in 42% (355/846) of patients with signs of envenomation (Figure 1). Among the envenomation cases, local complications (196/846, 23.2%) and systemic complications (290/846, 34.3%) were observed. Of the 355 patients with complications, hemorrhage (62.3%), bite-site wounds (51%), and shock (38.9%) were the most common. Death was reported at a frequency of 7.6% (27/355) (Figure 3).

### 3.3. Factors Associated with Snakebite Complications

Bivariate analysis showed that age, residence, comorbidity, incision at the anatomical bite site, tourniquet application, use of black stone, bleeding, and the presence of abnormal vital and neurological signs were significantly associated with complications of snakebite envenomation (Table 3). These variables, along with the administration of serum antivenom (*p* = 0.20), were included in the multivariate adjusted regression model. In the multivariate logistic regression, after adjustment, victims younger than 15 years (AOR: 2.04; 95% CI: 1.14–3.72), those aged 15–29 years (AOR: 1.87; 95% CI: 1.03–3.44), and those residing in rural areas (AOR: 4.80; 95% CI: 2.21–11.4) were significantly more likely to develop complications than their counterparts. The risk of developing complications after envenomation was five times higher when the local practice of incision at the anatomical site of the snakebite was performed (AOR: 4.31; 95% CI: 2.51–7.52), and tourniquet was applied (AOR: 5.52; 95% CI: 1.42–30.8). Patients with abnormal vital signs (AOR: 14.3; 95% CI: 9.22–22.7) and bleeding (AOR: 14.2; 95% CI: 8.80–23.4) had 14-fold higher odds of developing complications from snakebite envenomation. Patients who did not receive serum antivenom (AOR: 2.92; 95% CI: 1.80–4.80) were three times more likely to develop complications (Table 3).

After adjustment, variables such as sex, time of year of envenomation, comorbidity, application of black stone, presence of local signs, and presence of neurological signs were not associated with complications of snakebite envenomation.

## 4. Discussion

This study examined data on snakebite envenomation in the Cascades region of Burkina Faso to determine the frequency of complications and identify associated risk factors. With a sample size of 846 cases, our study significantly contributes to the epidemiological understanding of snakebite complications in Burkina Faso. Previous studies in the region have been limited to hospital-based case studies [12,13,14]. To improve prevention and management strategies, it is important to evaluate the factors associated with snakebite complications over an extended period.

The study found a relatively high frequency of snakebite complications, with 42% of the cases resulting in complications. Local complications, such as wounds and skin necrosis, were observed in 23% of patients. Systemic complications, including hemorrhage, shock, and coma, were observed in 34%. These often require immediate medical intervention. Mortality was observed in 7.6% of patients with complications. Common local practices may have intensified these complications. Bite-site incisions and tourniquet application were frequently reported in the study area. Black stone made from burned pieces of cow bone was applied as first aid after bite-site incision [19]. The results of the study showed that tourniquet application increased the risk of complications by a factor of 5.52. Local incisions increased it by a factor of 4.31. Worsened snakebite complications likely contributed to the observed mortality rate. It is essential to address not only the acute complications but also the chronic disabilities associated with snakebite envenomation, as observed in similar studies in Cameroon and Ghana. Immediate medical intervention is vital, but comprehensive follow-up is also essential for managing chronic disabilities and psychological trauma. Public health strategies should focus not only on improving the accessibility and quality of acute medical care but also on providing support for the long-term rehabilitation of people who have experienced snakebite complications [4,20].

The study also found that rural populations were nearly five times more likely to develop complications compared with urban populations. This could be explained by the frequent recourse to traditional medicine due to cultural proximity to traditional practitioners and the inaccessibility of modern health care in rural areas [21]. Victims often preferred traditional care, which includes harmful practices such as local incisions and tourniquets, both of which favor the onset of complications [4]. Similar practices and outcomes have been observed in Nigeria, Eswatini, and Ghana, highlighting the significant influence of traditional medicine on snakebite management in rural settings. This preference not only delays access to effective medical treatment but also increases the risk of severe complications. Therefore, public awareness of the risks associated with traditional practices and the accessibility and affordability of modern health care in rural settings are crucial [22,23,24].

The pediatric population was more susceptible and accounted for a significant proportion of envenomation cases (44%). Children under 15 and adults aged 15 to 30 years were twice as likely to develop complications as victims aged 30 and older. The severity of envenomation depends on the volume of venom injected and the degree to which it is absorbed. This may explain the vulnerability of children to complications, as they have a smaller body surface area and therefore a higher concentration of venom [25,26]. A previous study in South Africa confirmed that children are particularly vulnerable to local and systemic envenomation syndromes, which occur more frequently and with greater severity in this age group [27]. This finding underscores the need for targeted interventions to protect children who are at higher risk of severe outcomes because of their physiological characteristics and behavioral patterns, which might expose them to snakebite risks.

Bleeding from mucous membranes, recent wounds, or scars were common in the study sample, including cases of epistaxis, gingivorrhagia, hematemesis, hemoptysis, and a few cases of melena and hematuria. The presence of bleeding increased the risk of complications by 14. Snake venoms, particularly those from the *Viperidae* specie, contain enzymes that alter cell membranes, vascular walls, and blood coagulation cascades [28]. Bleeding related to coagulation disorders may have led to hypovolemic shock in this study. Increased vascular permeability due to snake venom could cause other complications, explaining abnormal vital signs. Hemoperitoneum, subdural hematoma, acute renal failure, and pulmonary edema have been previously reported [29]. The cardiotoxic effects of the venom, which were not analyzed in this study, may have contributed to complications or death [30].

Failure to administer serum antivenom significantly increased the risk of complications. Although antivenoms are available at some health facilities, their high cost makes them inaccessible to most victims. Early administration of antivenom is crucial for effective treatment [31]. Therefore, making antivenoms affordable and available in primary health care centers, especially in rural settings, is recommended. Additionally, harmful practices should be actively discouraged through public health campaigns and education programs. It is also crucial to promote safe and effective first aid practices. Traditional remedies with proven efficacy should be considered, particularly in rural areas where antivenoms are neither readily available nor affordable.

Our study has limitations due to its retrospective nature, which may introduce bias, particularly in terms of data completeness and accuracy. This study relied on the retrospective analysis of routine data obtained from consultations and medical records conducted by others, which was often incomplete. This meant that these records lacked detailed descriptions of the characteristics of people who experienced snakebite. Additionally, the absence of some independent variables from the data sources may have hindered a comprehensive understanding of other factors related to envenomation complications. We could not include the site of the bite as a variable, which might have provided more insight into the severity and progression of envenomation. Another significant limitation is the lack of detailed information on the species of snakes that bite. Because venom properties vary significantly between species, these missing data could affect the interpretation of clinical outcomes and efficacy of the antivenoms used. Despite these limitations, our results highlight important practical implications for improving health care in rural settings. Future prospective studies with comprehensive data collection on snake species and anatomical bite sites are needed to better elucidate these factors and improve the reliability of the findings.

## 5. Conclusions

This study provided baseline information on the factors associated with snakebite complications in the Cascades region of Burkina Faso. Significant factors included rural residence, young age, incision at the bite site, and tourniquet application. Bleeding and abnormal vital signs were also strongly linked to complications. The administration of serum antivenom significantly reduced these risks. To reduce complications, it is essential to increase the availability of serum antivenom in rural settings and to educate the population about seeking prompt medical care. Effective educational programs, such as radio broadcasts, can raise awareness about the dangers of traditional treatments and provide proper first aid for snakebites. Further prospective studies are needed to confirm these findings and explore other factors influencing envenomation complications, including chronic disabilities and long-term outcomes.

## Figures and Tables

**Table 1:** Sociodemographic characteristics of snakebite victims in the Cascades region over the period 2016–2021.

Characteristics	Number (*n* = 846)	Values (%)
Median age (IQR) ^a^	17 (10–32)	
Age range ^a^		
≥30	251	29.7
15–29	226	26.7
<15	369	43.6
Gender		
Male	462	54.6
Female	384	45.4
Residence		
Urban	100	11.8
Rural	746	88.2
Period of the year		
First half ^b^	416	49.2
Second half ^b^	430	50.8

^a^ Age in years. ^b^ First half: January–June. Second half: July–December. IQR, interquartile range.

**Table 2:** Clinical characteristics of snakebite envenomation victims in health facilities in the Cascades region of Burkina Faso, 2016–2021.

Characteristics	Number (*n* = 846)	Values (%)
Comorbidity		
Yes	50	5.9
No	796	94.1
Local incision		
Yes	201	23.8
No	645	76.2
Tourniquet		
Yes	67	7.9
No	779	92.1
Black stone		
Yes	118	13.9
No	728	86.1
Local signs		
Yes	811	95.9
No	35	4.1
Abnormal vital signs		
Yes	413	48.8
No	433	51.2
Bleeding/Hemorrhage		
Yes	275	32,5
No	571	67.5
Dyspnea		
Yes	95	11.2
No	751	88.8
Neurological signs		
Yes	53	6.3
No	793	93.7
Ophthalmological signs		
Yes	17	2.0
No	829	98.0
Serum antivenom		
Yes	590	69.7
No	256	30.3

**Table 3:** Factors associated with complications related to snakebite envenomation in the Cascades region of Burkina Faso over the period 2016–2021.

Features	OR ^a^	95% CI		*p*-Value	AOR	95% CI		*p*-Value
		Low	High			Low	High	
Gender				0.3				
Female	1							
Male	1.17	0.89	1.54					
Age (years)				0.01				0.047
≥30	1				1			
15–29	1.41	0.95	2.13		1.87	1.03	3.44	
<15	1.78	1.2	2.66		2.04	1.14	3.72	
Place of residence				<0.001				<0.001
Urban	1				1			
Rural	6.92	3.8	13.9		4.8	2.21	11.4	
Time of year				0.4				
First half ^b^	1							
Second half ^b^	0.9	0.68	1.18					
Comorbidity				0.003				
No	1							
Yes	2.38	1.33	4.35					
Local incision				<0.001				<0.001
No	1				1			
Yes	7.77	5.4	11.4		4.31	2.51	7.52	
Tourniquet				<0.001				0.011
No	1				1			
Yes	35.8	13.1	147		5.52	1.42	30.8	
Black stone				<0.001				
No								
Yes	2.46	1.66	3.69					
Local signs				0.8				
No	1							
Yes	1.09	0.55	2.22					
Abnormal vital signs				<0.001				<0.001
No	1				1			
Yes	16	11.4	22.9		14.3	9.22	22.7	
Bleeding				<0.001				<0.001
No	1				1			
Yes	14.3	10	20.6		14.2	8.8	23.4	
Neurological signs				<0.001				
No	1							
Yes	19.5	7.86	65					
Serum antivenom				0.2				<0.001
Yes	1				1			
No	0.8	0.59	1.08		2.92	1.8	4.8	

^a^ Unadjusted odd ratio. ^b^ First half: January–June and Second half: July–December. AOR: adjusted odd ratio. CI: confidence interval.

## Data Availability

The raw data supporting the conclusions of this article can be made available by the authors without a prior reservation.

## References

[1] Chippaux J.-P., Massougbodji A., Habib A.G. (2019). The WHO strategy for prevention and control of snakebite envenoming: A sub-Saharan Africa plan. J. Venom. Anim. Toxins Incl. Trop. Dis..

[2] Chippaux J.-P., Akaffou M.H., Allali B.K., Dosso M., Massougbodji A., Barraviera B. (2016). The 6th international conference on envenomation by Snakebites and Scorpion Stings in Africa: A crucial step for the management of envenomation. J. Venom. Anim. Toxins Incl. Trop. Dis..

[3] World Health Organization (2019). Snakebite Envenoming: A Strategy for Prevention and Control.

[4] Alcoba G., Chabloz M., Eyong J., Wanda F., Ochoa C., Comte E., Nkwescheu A., Chappuis F. (2020). Snakebite epidemiology and health-seeking behavior in Akonolinga health district, Cameroon: Cross-sectional study. PLoS Negl. Trop. Dis..

[5] Touré M., Coulibaly M., Koné J., Diarra M., Coulibaly B., Beye S., Diallo B., Dicko H., Nientao O., Doumbia D. (2019). Complications aigues de l’envenimation par morsures de serpent au service de réanimation du CHU mère enfant “LE Luxembourg” de Bamako. Mali. Méd..

[6] Chippaux J.-P. (2011). Estimate of the burden of snakebites in sub-Saharan Africa: A meta-analytic approach. Toxicon.

[7] Gampini S., Nassouri S., Chippaux J.-P., Semde R. (2016). Retrospective study on the incidence of envenomation and accessibility to antivenom in Burkina Faso. J. Venom. Anim. Toxins Incl. Trop. Dis..

[8] Bamogo R., Nikièma A.S., Belem M., Thiam M., Diatta Y., Dabiré R.K. (2023). Cross-sectional ethnobotanical survey of plants used by traditional health practitioners for snakebite case management in two regions of Burkina Faso. Phytomed. Plus.

[9] Sorge F., Chippaux J.P. (2016). Prise en charge des morsures de serpent en Afrique. Lett. L’infect..

[10] Bamogo R., Thiam M., Nikièma A.S., Somé F.A., Mané Y., Sawadogo S.P., Sow B., Diabaté A., Diatta Y., Dabiré R.K. (2021). Snakebite frequencies and envenomation case management in primary health centers of the Bobo-Dioulasso health district (Burkina Faso) from 2014 to 2018. Trans. R. Soc. Trop. Med. Hyg..

[11] Ahmed S., Koudou G.B., Bagot M., Drabo F., Bougma W.R., Pulford C., Bockarie M., Harrison R.A. (2021). Health and economic burden estimates of snakebite management upon health facilities in three regions of southern Burkina Faso. PLoS Negl. Trop. Dis..

[12] Ouédraogo P.V., Traoré C., Savadogo A.A., Bagbila W.P.A.H., Galboni A., Ouédraogo A., Sere I.S., Millogo A. (2022). Hémorragie cérébro-méningée secondaire à une envenimation par morsure de serpent: À propos de deux cas au Centre Hospitalier Universitaire Sourô Sanou de Bobo-Dioulasso, Burkina Faso. Med. Trop. Sante Int..

[13] Dabilgou A.A., Sondo A., Dravé A., Diallo I., Kyelem J.M.A., Napon C., Kaboré J. (2021). Hemorrhagic stroke following snake bite in Burkina Faso (West Africa). A case series. Trop. Dis. Travel. Med. Vaccines.

[14] Kyelem C., Yaméogo T., Ouédraogo S., Zoungrana J., Poda G., Rouamba M., Ouangré A., Kissou S., Rouamba A. (2012). Snakebite in Bobo-Dioulasso, Burkina Faso: Illustration of realities and challenges for care based on a clinical case. J. Venom. Anim. Toxins Incl. Trop. Dis..

[15] Chippaux J.-P. Les serpents d’Afrique occidentale et centrale. Éditions de l’IRD (EX-ORSTOM). 2001;310. https://www.editions.ird.fr/produit/232/9782709920001/les-serpents-d-afrique-occidentale-et-centrale.

[16] Chirio L. (2009). Inventaire des reptiles de la région de la Réserve de Biosphère Transfrontalière du W (Niger/Bénin/Burkina Faso: Afrique de l’Ouest). Bull. Soc. Herp. Fr..

[17] National Institute of Statistics and Demography (2022). Fifth General Census of Population and Housing in Burkina Faso. https://burkinafaso.opendataforafrica.org.

[18] Chippaux J.-P. (2015). Management of Snakebites in Sub-Saharan Africa. Méd. Santé Trop..

[19] Francis M.F., Vianney J.-M., Heitz-Tokpa K., Kreppel K. (2023). Risks of snakebite and challenges to seeking and providing treatment for agro-pastoral communities in Tanzania. PLoS ONE.

[20] Aglanu L.M., Amuasi J.H., Schut B.A., Steinhorst J., Beyuo A., Dari C.D., Agbogbatey M.K., Blankson E.S., Punguyire D., Lalloo D.G. (2022). What the snake leaves in its wake: Functional limitations and disabilities among snakebite victims in Ghanaian communities. PLoS Negl. Trop. Dis..

[21] Mhaskar D., Agarwal A., Bhalla G. (2014). A study of clinical profile of snake bite at a tertiary care centre. Toxicol. Int..

[22] Steinhorst J., Aglanu L.M., Ravensbergen S.J., Dari C.D., Abass K.M., Mireku S.O., Adu Poku J.K., Enuameh Y.A.K., Blessmann J., Harrison R.A. (2021). ‘The medicine is not for sale’: Practices of traditional healers in snakebite envenoming in Ghana. PLoS Negl. Trop. Dis..

[23] Ndu I., Edelu B., Ekwochi U. (2018). Snakebites in a Nigerian children Population: A 5-year review. Sahel. Med. J..

[24] Nann S. (2021). How beliefs in traditional healers impact on the use of allopathic medicine: In the case of indigenous snakebite in Eswatini. PLoS Negl. Trop. Dis..

[25] Gerardo C.J., Vissoci J.R.N., Evans C.S., Simel D.L., Lavonas E.J. (2019). Does This Patient Have a Severe Snake Envenomation?: The Rational Clinical Examination Systematic Review. JAMA Surg..

[26] Essafti M., Fajri M., Rahmani C., Abdelaziz S., Mouaffak Y., Younous S. (2022). Snakebite envenomation in children: An ongoing burden in Morocco. Ann. Med. Surg..

[27] Wagener M., Naidoo M., Aldous C. (2017). Wound infection secondary to snakebite. S. Afr. Med. J..

[28] Sajeeth Kumar K., Narayanan S., Udayabhaskaran V., Thulaseedharan N. (2018). Clinical and epidemiologic profile and predictors of outcome of poisonous snake bites &ndash; an analysis of 1500 cases from a tertiary care center in Malabar, North Kerala, India. Int. J. Gen. Med..

[29] Coulibaly M., Mangane M.I., Ouedrago Y., Koita S.A., Madane D.T., Hamidou A.A., Nientao O., Bagayogo D.K., Dembele A.S., Djibo D.M. (2021). Overview of poisoning by snake bite in 2019 at UHC Gabriel Touré of Bamako: Clinical features, prognosis and evaluation of the availability of antivenom serum. Méd. Intensive Réanim..

[30] Harikrishnan M.P., Anil Kumar C.R., Anand M.K., Earali J. (2021). Effects of hemotoxic snake bite envenomation on haematological parameters variability in predicting complications. Int. J. Med. Med. Res..

[31] Al Masroori S., Al Balushi F., Al Abri S. (2022). Evaluation of Risk Factors of Snake Envenomation and Associated Complications Presenting to Two Emergency Departments in Oman. Oman Med. J..

